# Supplementary figures and images for: Attentional Control via Parallel Target-Templates in Dual-Target Search
Source: PLoS One. 2014 Jan 28;9(1):e86848. doi: 10.1371/journal.pone.0086848 (PMC3904919; doi:10.1371/journal.pone.0086848)

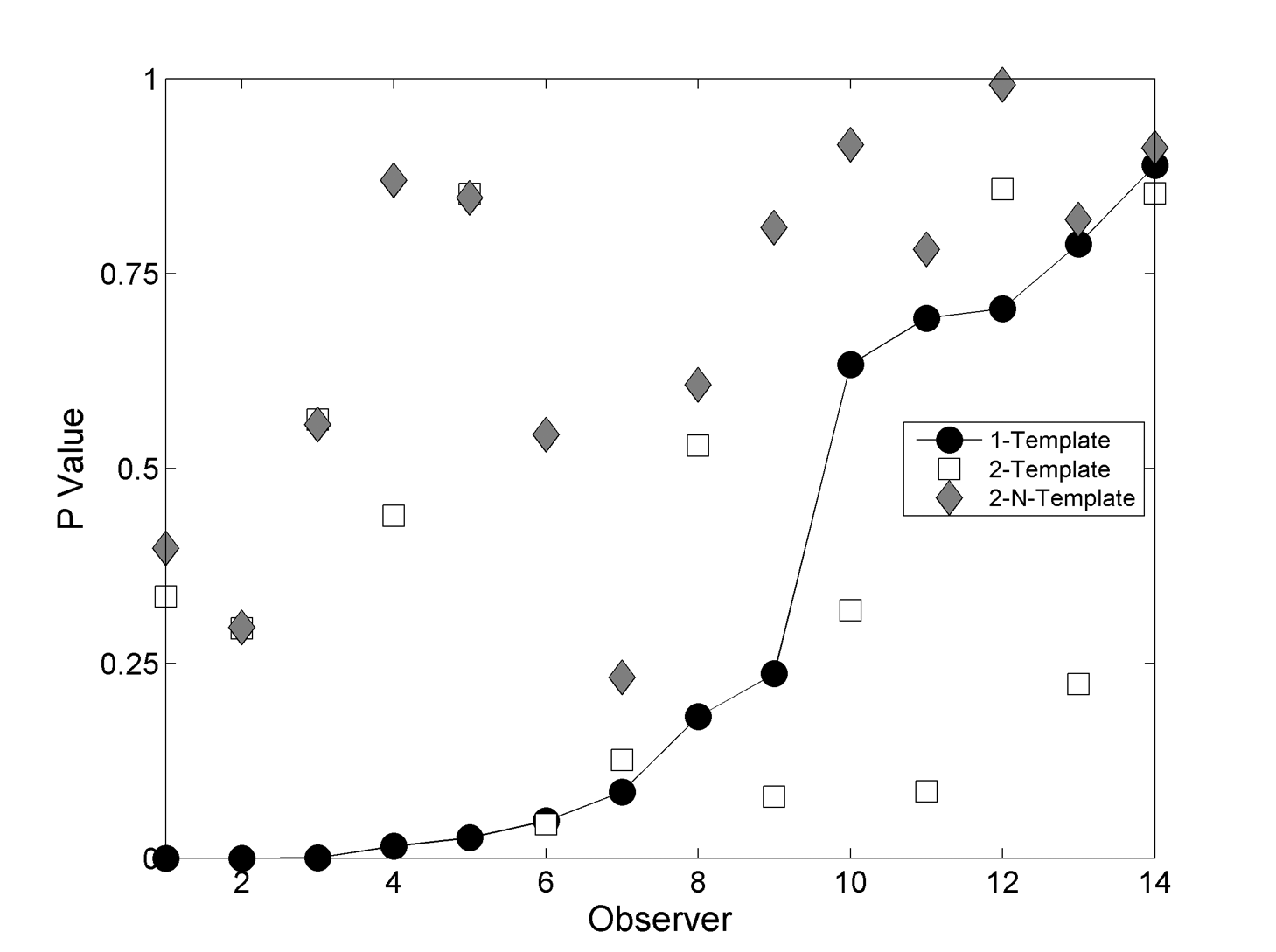

Supplement: Figure S1 — Individual P values for a X2 goodness-of-fit test for the 1-Template, 2-Template and 2-Noisy-Template models of single- and dual-target search. (TIF) [file pone.0086848.s001.tif]
