# Supplementary material for: Correction: Attentional Control via Parallel Target-Templates in Dual-Target Search
Source: PLoS One. 2014 Apr 4;9(4):e94894. doi: 10.1371/journal.pone.0094894 (PMC3976394; doi:10.1371/journal.pone.0094894)
Supplement: File S2 — Republished corrected article. (PDF) [file pone.0094894.s002.pdf]

OPEN ACCESS Freely available online

PLOS ONE

Attentional Control via Parallel Target-Templates in Dual-Target Search

Doug J. K. Barrett[1]*, Oliver Zobay[2]

1 School of Psychology, College of Biological and Medical Science, University of Leicester, Leicester, United Kingdom, 2 Medical Research Council Institute of Hearing Research, Nottingham, United Kingdom

Abstract

Simultaneous search for two targets has been shown to be slower and less accurate than independent searches for the same two targets. Recent research suggests this 'dual-target cost' may be attributable to a limit in the number of target-templates than can guide search at any one time. The current study investigated this possibility by comparing behavioural responses during single- and dual-target searches for targets defined by their orientation. The results revealed an increase in reaction times for dual- compared to single-target searches that was largely independent of the number of items in the display. Response accuracy also decreased on dual- compared to single-target searches: dual-target accuracy was higher than predicted by a model restricting search guidance to a single target-template and lower than predicted by a model simulating two independent single-target searches. These results are consistent with a parallel model of dual-target search in which attentional control is exerted by more than one target-template at a time. The requirement to maintain two target-templates simultaneously, however, appears to impose a reduction in the specificity of the memory representation that guides search for each target.

Citation: Barrett DJK, Zobay O (2014) Attentional Control via Parallel Target-Templates in Dual-Target Search. PLoS ONE 9(1): e86848. doi:10.1371/journal.pone.0086848

Editor: Joy J. Geng, University of California, Davis, United States of America

Received September 12, 2013; **Accepted** December 16, 2013; **Published** January 28, 2014

Copyright: © 2014 Barrett, Zobay. This is an open-access article distributed under the terms of the Creative Commons Attribution License, which permits unrestricted use, distribution, and reproduction in any medium, provided the original author and source are credited.

Funding: This work was supported by the University of Leicester. The funders had no role in study design, data collection and analysis, decision to publish, or preparation of the manuscript.

Competing Interests: The authors have declared that no competing interests exist.

* E-mail: djkb1@le.ac.uk

Introduction

As you rushed to catch your bus this morning, you may have been delayed by a last minute search for your keys *and* your phone. While this may have imposed a frustrating delay on your journey, the ability to search for multiple targets can have more important consequences in safety critical situations. Checking baggage for a gun or the components of an explosive device, for example, requires security personnel to find multiple targets in complex visual displays. Previous research has shown that this task is difficult: dual-target search is often slower and less accurate than separate searches for the same two targets. This 'dual-target cost' is observed when the targets are differentiated from non-targets (distractors) by values along a single feature-dimension (e.g., their colour or orientation [1–2]) and by variation across a range of feature-conjunctions (e.g., colour and orientation [3–4]). The magnitude of the dual-target cost depends upon the similarity of the two targets as well as their relation to the distractors: targets occupying non-contiguous regions within a feature-dimension elicit a larger dual-target cost as the distance between them and the number of intervening distractors increases [1–2]. According to a number of models, memory-directed search requires observers to compare objects in the scene with a mental representation of the target [5], [6]. In a single-target search, this 'target-template' is thought to provide a top-down bias for visual neurons that respond to the target's features in the display [7]. When more than one target is sought, interactions between the information maintained in memory and visual input must be distributed across multiple objects, increasing competition for memory and attentional resources [8], [9]. How the brain resolves this competition during search, and its effect on target detection, have yet to be fully determined.

Previous research suggests that competition for attentional control during search may be resolved in one of two ways: via a parallel process that is informed by simultaneously active target-templates [10], [11] or a serial process, that restricts attentional control at any one time to a single target-template [12], [13]. According to the serial account, the functional status of items in memory is determined by the current focus of attention (e.g., [14], [15]). Active items, such as the target-template during search, must be available to guide the cognitive operations required to differentiate the target from distractors. Items outside the current focus of attention are maintained in a passive state, which neither contributes towards, nor interferes with, the search process. This functional distinction imposes a temporal cost on dual-target search because observers are required to switch the status of consecutive target-templates to search for both targets [16], [17]. Evidence to support this model was reported in a study that used a rapid serial visual presentation (RSVP) task to compare observers' accuracy when they searched for one or two targets [12]. The results revealed a reliable decrease in accuracy on dual- compared to single-target searches, with performance best described by a signal detection (SDT) model that limited attentional control to a single target-template at any one time. According to this, the dual-target cost in single-fixation displays arises because observers are unable to switch the status of the target-templates in VWM quickly enough to search for both targets.

The serial account of the dual-target cost in RSVP tasks provides a potential explanation for the dual-target costs reported in longer duration free-view tasks (e.g., [1–4]). Increased response times (RTs) on dual- compared to single-target searches could be caused by the serial activation of consecutive target-templates. Within the SDT framework, decreases in accuracy might also be attributed to successive single-target searches: the probability of a false alarm during a dual-target search is expected to be higher than that for a single-target search because the comparison of each target-template with visual input generates an independent source of error [18–20]. Based on the assumption that objects in the display elicit noisy representations that are compared against an internal decision standard or criterion, the SDT models used by Houtkamp and Roelfsema [12] provide an estimate of the decrease in accuracy that can be attributed to stochastic noise when observers are required to conduct consecutive single-target searches. In the serial account, the assumption of independence is explicit, because attentional control at any one time is exclusive to the active target-template. Recent findings, however, indicate this temporal exclusivity may not characterise dual-target search. Roper and Vecera [11] and Irons and colleagues [21] used cued RSVP tasks to elicit single- and dual-target searches for targets defined by one or two colours. Spatial distractors that preceded the target produced an attentional blink on single- and dual-target searches, but only when they shared the same colour as the cue(s). This finding suggests dual-target searches were guided by simultaneously active target-templates, a finding that is difficult to reconcile with the serial account of attentional control.

The possibility that observers simultaneously activate two target-templates during search provides an alternative to the serial account of the dual-target cost. Data from Change Detection tasks has shown that the precision of items in VWM decreases as a function of the number of to-be-remembered objects [22–24]. Generalising this finding to search, predicts a reduction in the quality of the information used to categorise objects in the display when observers have to remember more than one object. Solomon and colleagues recently reported evidence consistent with this prediction in a task designed to investigate interactions between selective attention and VWM [25]. Adding a memory load during search increased overall RTs but did not affect the slopes of RT-by-set-size functions. Eye movements were also less accurate on searches that included a concurrent memory load, with observers less likely to fixate targets in the display. A similar effect has been observed during dual-target searches, with observers more likely to fixate objects dissimilar to the targets on dual- compared to single-target searches [2]. In the latter, the decrease in the accuracy of eye movements might reflect a change in search strategy, with observers optimising scan paths to identify two rather than one target. In Solomon et al.'s study, however, this should not have occurred because the items in VWM were irrelevant to the search. Taken together, these results support a decrease in the quality of the information available to guide search when observers are required to distribute attention to multiple objects in VWM. Intriguingly, Solomon et al.'s results (see also [35]) show this reduction has very little influence on the speed with which search proceeds through the display. This finding suggests the simultaneous activation of two target-templates may reduce target discriminability without incurring a concomitant increase in RT-by-set-size slopes on dual- compared to single-target searches.

The findings above support alternate explanations of the dual-target cost. Interpreting these is difficult because the data were obtained using different experimental tasks and measures. The SDT models used by Houtkamp and Roelfsema [12] are typically used in single-fixation displays, which are unusual in the real world. In information theoretic terms, the reduction of uncertainty during search is dynamic, with the interaction between visual input and the information in VWM developing over time [26]. Attentional control in RSVP tasks is also applied to one perceptual object at a time, while attentional control during search operates to prioritise one (or more) of a number of simultaneously presented objects for further analyses. Single-fixation displays, therefore, may elicit different search strategies than the free-view displays used to investigate accuracy and eye movements during dual-target search (e.g., [1–3]). Inferring changes in the amount of information available to guide single- and dual-target search using eye movements is also difficult: the probability of fixating a particular object is likely to reflect strategic responses to changes in the discriminability of the target as well as the decision criteria against which visual input is compared. Adopting a more conservative response criterion, for example, is likely to affect the number of objects fixated during search, with observers requiring more information to accept the presence of a target before they terminate the search. Finally, differences in the perceptual properties of the targets in Houtkamp and Relfsema's study may have elicited a serial search strategy that prioritised the more discriminable target first [1].

To address these concerns, the current experiment was designed to distinguish between the serial and parallel accounts of the dual-target cost in free-view displays. To do this, we examined SDT measures of accuracy and RT as a function of set size to answer three questions: (a) is dual-target search characterised by a reduction in the amount of information available to guide search, (b) does any decrease in accuracy on dual-target searches exceed that predicted by the increase in stochastic noise generated by two independent single-target searches over one single-target search and (c) is any slowing of RTs as a function of set size consistent with two consecutive single-target searches? The SDT framework provides a means of modelling dual-target accuracy when the amount of information available to guide search varies between one and two target-templates. SDT analyses can also be used to differentiate dual-target costs attributable to changes in target discriminability, the observer's response criterion, and the increase in stochastic noise associated with two independent single-target searches. In free-view displays, this information can be assessed in terms of the time-course of the search process. More specifically, comparisons of the RT-by-set-size slopes for single- and dual-target searches provide an explicit test of the possibility that observers conduct consecutive single-target searches when two targets are sought.

Method

Participants

Fourteen undergraduate students attending the University of Leicester were recruited to the study (M_{age} 22.4, range: 19–27 years). All reported normal or corrected-to-normal vision and were awarded research credits for their participation. All experimental procedures conformed to the Code of Ethics of the World Medical Association (Declaration of Helsinki) and approval for the study was obtained from the School of Psychology's Ethics Committee at the University of Leicester. Observers provided written consent prior to their participation.

Apparatus

The experiment was run on an IBM PC with a 19′ View Sonic G90fB monitor (Walnut, CA, USA). The display resolution was 1240×768 pixels and the frame rate was 85 Hz. The experiment was controlled using custom-build software in MATLAB (Math-

works, Natick, MA, USA) with Psychophysics toolbox extensions [27]. Viewing distance was maintained at 57 cm using a fixed chin rest and responses were collected using a Cedrus RB-350 Response Pad (San Pedro, CA, USA). The experiment was conducted in a quiet, dimly lit room.

Stimuli

The task used orientation stimuli that were designed to: (a) equate the discriminability of two targets that could be separately cued, (b) elicit variation in the accuracy of responses and RTs as a function of set size and (c) maximise competitive interactions within VWM by requiring observers to search for targets defined by different values from a single feature-dimension [24], [28]. Displays contained rectangular stimuli that subtended 2.5° by 0.5° of visual angle. The rectangles were dark grey in colour (24 cd/m^2) and were presented on a uniform mid grey background (44 cd/m^2). Displays could contain 4, 8, 12 or 16 rectangles that were randomly assigned a location within two 3×5 virtual grids centred in the left and right visual fields at an eccentricity of 6.75°. The two grids abutted at the vertical midline and grid elements subtended 4.5 by 4.5 degrees. Rectangles on each trial were displaced from the centre of each element by a randomly generated value. Target rectangles were always oriented at 45° and 135° and distractors were oriented at 15°, 75°, 105 and 165°, with each target designed to group with its neighbouring distractors in separate upward and downward tilting groups. Equal numbers of rectangles from each group were assigned to the left and right visual fields on each trial to ensure an even distribution of both groups across the vertical midline.

Procedure

The experiment used a factorial design to manipulate four independent variables: search type (single- or dual-target), trial type (target-present or target-absent), target identity (Target 1 or 2), and set size (4, 8, 12, and 16). Experimental blocks contained four repetitions of this structure (128 trials) presented in a randomised sequence and observers completed a total of 5 experimental blocks (640 trials in total). On trials with a set size of four, target-absent displays contained one rectangle at each of the orientations assigned to the four distractors. For set sizes of 8, 12 and 16, the number of rectangles at each orientation was multiplied by 2, 3 and 4 respectively. On target-present trials, the search display always contained one rectangle oriented at the value assigned to Target 1 *or* Target 2. Targets always replaced one of the distractors from the same group (i.e., ±30°).

Figure 1 illustrates the sequence of events on each trial. Trials began with a fixation-cross at the centre of the screen. After 750 ms, a cue containing a rectangle above and below the central fixation was presented. On single-target trials, both rectangles were presented at the orientation assigned to Target 1 *or* Target 2. On dual-target trials, the rectangles were presented at two different orientations, those assigned to Target 1 *and* Target 2. Single- and dual-target search trials were cued with an equal probability and the position of the rectangles on dual-target cues was randomly assigned. Cues remained on the screen for 1000 ms and were followed by an inter-trial-interval (ISI) of 1000 ms before the onset of the search display. Participants were then required to make a speeded response indicating whether a cued rectangle (target) had appeared in the search display. Once a response had been recorded, written feedback was presented to the centre of the screen.

Signal Detection Models of Accuracy

Following the method of Houtkamp and Roelfsema [12], the proportion of hits and false alarms on single- and dual-target searches were compared with those predicted by three generalisations of an equal-variance SDT model of single-target search. These assume information about each object in the display is registered in a separate channel or perceptual filter and integrated via an independent decisions mechanism [29]. In the equations in Table 1, the sub-indices s and d refer to single- and dual-target searches and f and h the predicted false alarm and hit rates respectively. Φ denotes the cumulative normal distribution function, d' the derived index of target discriminability, λ the response criterion and n is set size.

On a single-target search, the models above assume the number of comparisons required to (correctly or erroneously) reject the presence of a target is equivalent to the number of objects in the display (n). As false alarms occur with distractors, the probability of a false alarm when the target is absent is $1–\Phi(\lambda)^n$, with $\Phi(\lambda)^n$ describing the probability of correctly rejecting the target at n locations in the display. When the target is present, the probability of a target-absent response equals the product of the probabilities of missing the target ($\Phi(\lambda - d')$) and correctly rejecting the target at n –1 locations in the display ($\Phi(\lambda)^{n-1}$). The probability of a hit is thus $1–\Phi(\lambda - d')* \Phi(\lambda)^{n-1}$. These equations predict an increase in the number of false alarms as well as hits as set size increase from 4 to 16. Empirically, hit rates decrease with set size, and SDT models accommodate this finding by assuming observers adopt a progressively more conservative criterion as the number of comparisons required to detect or reject the target increases (i.e., λ increases as a function of n; see [30]). In extending this assumption to dual-target searches, we predict the requirement to compare each object with both target-templates will elicit a further

Table 1. Expressions for the 1-Template, 2-Template and 2-Noisy-Template models of single- and dual-target search.

1-Template	$f_s = 1 - \Phi(\lambda_{n,s})^n$	$f_d = 1- \Phi(\lambda_{n,d})^n$	(E.1)
9 parameters:	$h_s = 1 - \Phi(\lambda_{n,s} - d') * \Phi(\lambda_{n,s})^{n-1}$	$h_d = 0.5 * (1 - \Phi(\lambda_{n,d} - d') *$	
$\{\lambda_{n,s}\}, \{\lambda_{n,d}\}, d'$		$\Phi(\lambda_{n,d})^{n-1}) + 0.5 *(1 - \Phi(\lambda_{n,d})^n)$	
2-Template	$f_s = 1 - \Phi(\lambda_{n,s})^n$	$f_d = 1 - \Phi(\lambda_{n,d})^{2n}$	(E.2)
9 parameters	$h_s = 1 - \Phi(\lambda_{n,s} - d') * \Phi(\lambda_{n,s})^{n-1}$	$h_d = 1 - \Phi(\lambda_{n,d} - d') * \Phi(\lambda_{n,d})^{2n-1}$	
$\{\lambda_{n,s}\}, \{\lambda_{n,d}\}, d'$			
2-Noisy-Template	$f_s = 1 - \Phi(\lambda_{n,s})^n$	$f_d = 1 - \Phi(\lambda_{n,d})^{2n}$	(E.3)
10 parameters:	$h_s = 1 - \Phi(\lambda_{n,s} - d'_s) * \Phi(\lambda_{n,s})^{n-1}$	$h_d = 1 - \Phi(\lambda_{n,d} - d'_d) * \Phi(\lambda_{n,d})^{2n-1}$	
$\{\lambda_{n,s}\}, \{\lambda_{n,d}\}, d'_s, d'_d$			

doi:10.1371/journal.pone.0086848.t001

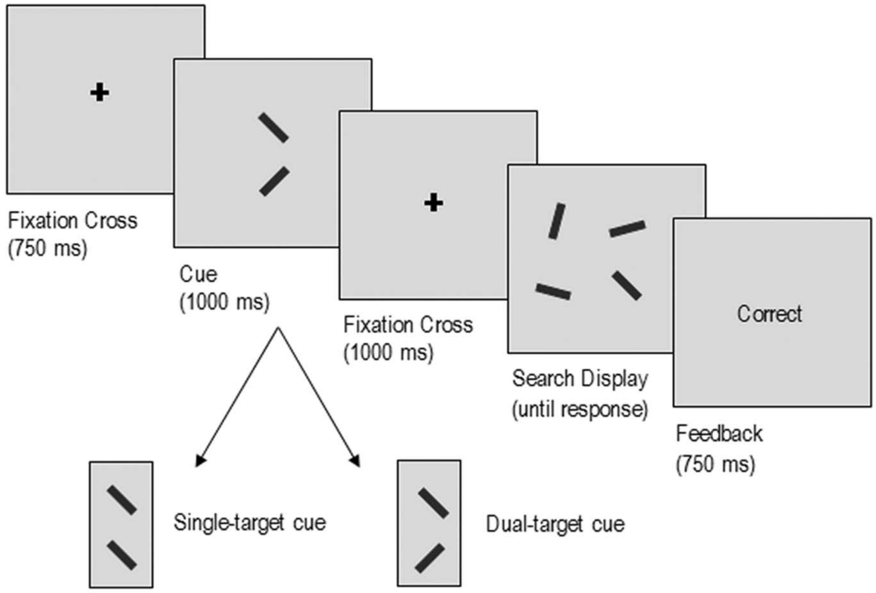

Figure 1. Sequence of events on a single trial with a set size of four. In this example, the cue signals a dual-target search on a target-present trial.
doi:10.1371/journal.pone.0086848.g001

increase in the decision criterion on dual- compared to single-target searches (i.e., $\lambda_{n,s}<\lambda_{n,d}$).

According to the one-template model (E.1) dual-target search is limited because the observer is only able to select and compare visual input to the information maintained in a single target-template. This simulates a situation in which the observer is either unable to switch between target-templates or terminates the search before the second target-template is activated. When the target in the display matches the active target-template, the one-template model predicts equivalent accuracy on single- and dual-target searches. When the target in the display matches the passively maintained target-template, however, the model predicts an absent response, because there is no information available to guide selection or the comparison process. Accordingly, the probability of a hit on a dual-target trial is the mean of the probabilities of a hit when the target matches the active target-template ($1-\Phi(\lambda_{n,d}-d') * \Phi(\lambda_{n,d})^{n-1}$) and a false alarm when it matches the passive target-template ($1-\Phi(\lambda_{n,d})^{n}$).

The two-template model (E.2), in contrast, assumes the observer is able to use both target-templates to guide selection and categorise objects in the display as targets or distractors. Discriminability for each target in the two-template model is equivalent to that on a single-target search but accuracy is expected to fall because the likelihood of an error for two independent processes is greater than that for either process alone [12], [20]. In this case, d' is constant but the exponent changes to $2n$–1 and $2n$ on target-present and target-absent searches respectively, to reflect a doubling of the number of comparisons required. Both models assume that: (a) increasing the number of target-distractor comparisons will increase the probability that a distractor will be confused with the target and (b) that observers are likely to adopt a more conservative criterion in response to increasing stochastic noise. Each model, however, makes a different prediction about the accuracy of dual-target search based upon the amount of information available to categorise objects in the display as a target or distractor.

The one- and two-template models both assume target discriminability (d') in a dual-target search is equivalent to that in a single-target search. Recent research, however, has revealed that increasing the number of objects in VWM reduces the precision with which they are maintained [22–24]. In the standard signal detection framework, d' is determined by two parameters; the distance between the distributions associated with the target and the distractors and the common standard deviation of these distributions [31]. If maintaining a second target-template during search leads to an increase in the variance of the target and distractor distributions, the result would be a decrease in d' on dual- compared to single-target searches. In order to test this possibility, the two-template model above was adapted to specify a 'two-noisy-template' model in which d' could vary on single- and dual-target searches (E.3).

To accommodate likely changes in the observers' response criteria, λ was allowed to vary across set size and search type in each of the models above. This produced 9 free parameters (d', λ_{4s} to λ_{16s} and λ_{4d} *to* λ_{16d}) in the one- and two-template models, and 10 in the two-noisy-template model (d'_s, d'_d, λ_{4s} to λ_{16s} and λ_{4d} *to* λ_{16d}). Maximum likelihood estimates (MLEs) of the parameters λ and d' were calculated for the observed hit and false alarm rates for each observer and compared using the Akaike information criterion (AIC) [32]. In information-theoretic terms, the AIC generates a relative estimate of each SDT model's ability to predict the observed pattern of results that is based on the number of free parameters and the log likelihood obtained. For each estimated SDT model, the distribution of the Pearson statistic X^2 was computed using the parametric bootstrap technique to assess goodness-of-fit [33]. By determining where the observed X^2 falls within this distribution, a p-value was calculated to provide a measure of the probability of the observed (or more extreme) frequency distributions under the estimated SDT model.

RT-by-set-size Functions

Median RTs for correct responses were used to calculate individual RT-by-set-size slopes and intercepts for each search and trial type. According to the serial account of the dual-target cost, attentional control at any one time is limited to a single target-

template. On dual-target searches, this imposes an RT cost as the status of separate target-templates in VWM is switched from active to passive [16–17]. When the target in the display matches the active target-template, the serial model predicts equivalent single- and dual-target slopes. When the target in the display matches the passively maintained target-template, the serial model predicts an increase in the slope, because the observer must conduct an exhaustive target-absent search before switching to the relevant target-template. This means target-present slopes for dual-target searches will increase by approximately half the single-target absent slope, because half the trials include a target-absent search. Two consecutive single-target-absent searches will also take twice as long as a single-target-absent search [3]. In free-view displays, these predictions provide an explicit test of the serial account of the dual-target search. Equation 4 predicts RT-by-set-size slopes for two consecutive, self-terminating searches, where p and a represent the slopes on target-present and target-absent trials and the sub-indices 1 and 2 denote the number of targets sought.

$$\text{Consecutive single-target searches}: a_2 = a_1 * 2 \qquad (4)$$
$$p_2 = p_1 + a_1/2$$

Results

Initial analyses to compare search performance for Targets 1 and 2 revealed comparable accuracy and RTs at each set size (all p's>0.05). Data for both targets were, therefore, collapsed into a search type (single- or dual-target), by trial type (target-present or absent) by set size (4, 8, 12, and 16) factorial design.

Accuracy

Table 2 presents the mean proportion of correct responses by search type and set size. As can be seen, single-target searches were more accurate than dual-target searches and accuracy for both types of search decreased as a function of set size. A 2×4 ANOVA on the proportion of correct scores with search type and set size as within-subjects factors yielded main effects of search type [$F(1,13) = 48.86$, $p<0.001$, $\eta_p^2 = 0.79$] and set size [$F(3,39) = 43.51$, $p<0.001$, $\eta_p^2 = 0.77$] but no significant Search type by Set size interaction [$F(3,39) <1$]. The data, therefore, reveal a reliable dual-target cost in accuracy that is independent of that associated with set size.

Figure 2 plots the mean observed against the mean predicted proportion of hits and false alarms for each SDT model by search type and set size. For single-target searches (left column), all SDT models produced a reasonable fit between the observed and predicted values. For dual-target searches, however, the fits vary across models (right column). For the one-template model, (1st row), the predicted values underestimate the proportion of observed hits and overestimate the proportion of false alarms across all four set sizes. For the two-template and two-noisy-template models, the fit between the observed and predicted hits and false alarms is much closer (2nd and 3rd row respectively).

Table 2. Mean proportion of correct responses for single- and dual-target searches by set size.

	Single-target search		Dual-target search	
Set size	Mean	SD	Mean	SD
4	.90	.07	.81	.10
8	.82	.09	.72	.12
12	.77	.11	.69	.10
16	.78	.10	.67	.11

doi:10.1371/journal.pone.0086848.t002

The increased fit between the observed and predicted accuracy for the two-template compared to the single-template model in Figure 2 is supported by the statistical comparisons listed in Table 3. For the one-template model, the summed AIC value is higher than for either of the two-template models, indicating a greater loss of information when the probability distribution of hits and false alarms is modelled using a single target-template. Across observers, goodness-of-fit p values for the one-template model varied between 0.89 and 0.00, with a value less than 0.05 for six observers indicating an extremely poor fit between the observed and predicted frequency distribution of hits and false alarms (see Figure S1).

For the two-template models, the summed AIC value was smallest for the two-noisy-template model where d' was free to vary by search type [single-target d', $M = 2.95$, dual-target d', $M = 2.53$; $t(13) = 3.83$, p = 0.002]. This advantage was also represented at the individual level where AIC values were smaller for 11 of the 14 observers. Goodness-of-fit p values were also higher for the two-noisy-template ($M = 0.68$) than the two-template model ($M = 0.40$). This pattern of results represents two important findings; first, observers are able to use information from more than one target-template to guide dual-target search and second, the reduction in accuracy on dual- compared to single-target searches is most likely to reflect a concomitant reduction in d'. Importantly, this indicates the dual-target cost exceeds that predicted by the increase in stochastic noise associated with two independent single-target searches, as well as the tendency for observers to adopt more conservative criteria as the number of comparisons required to detect or reject the target increases with set size and the number of targets sought (see Table 3).

RT Data

Table 4 presents mean RT-by-set-size slopes and intercepts for single- and dual-target searches. Figure 3 plots mean RTs for each search type and trial type by set size. As can be seen, slopes for target-present trials were shallower than those for target-absent trials on single- and dual-target searches, with the differences approximating the 1:2 ratio associated with inefficient search [34].

A 2×2 ANOVA on individual RT-by-set-size slopes with search type and trial type as within-subjects factors yielded main effects of search [$F(1,13) = 7.37$, $p = 0.018$, $\eta_p^2 = 0.362$] and trial type [$F(1,13) = 54.52$, $p<0.001$, $\eta_p^2 = 0.80$], but no significant Search type by Trial type interaction [$F(1,13) <1$]. The rate of search for dual- compared to single-target searches decreased, with slopes for target-absent trials steeper than those for target-present trials across both types of search. To compare the increase in slopes on dual- compared to single-target searches with those predicted by consecutive single-target searches, separate t-tests were conducted on the target-present and target-absent slopes for each type of search (see E.4 and Figure 3). The results revealed smaller observed than predicted increases on target-present [M difference = 61.79 ms, $SD = 47.42$ ms; $t(13) = 4.88$, $p<0.001$ for test of $p_1 + a_1/2 - p_2 = 0$] and target-absent [M difference = 140.67 ms, $SD = 56.25$ ms; $t(13) = 9.36$, $p<0.001$ for test of $a_1 * 2 - a_2 = 0$] trials. On target-absent trials, the increase in the dual-target slope represents just 17% of the time required to conduct a second single-target search, or less than one additional object at a set size of four.

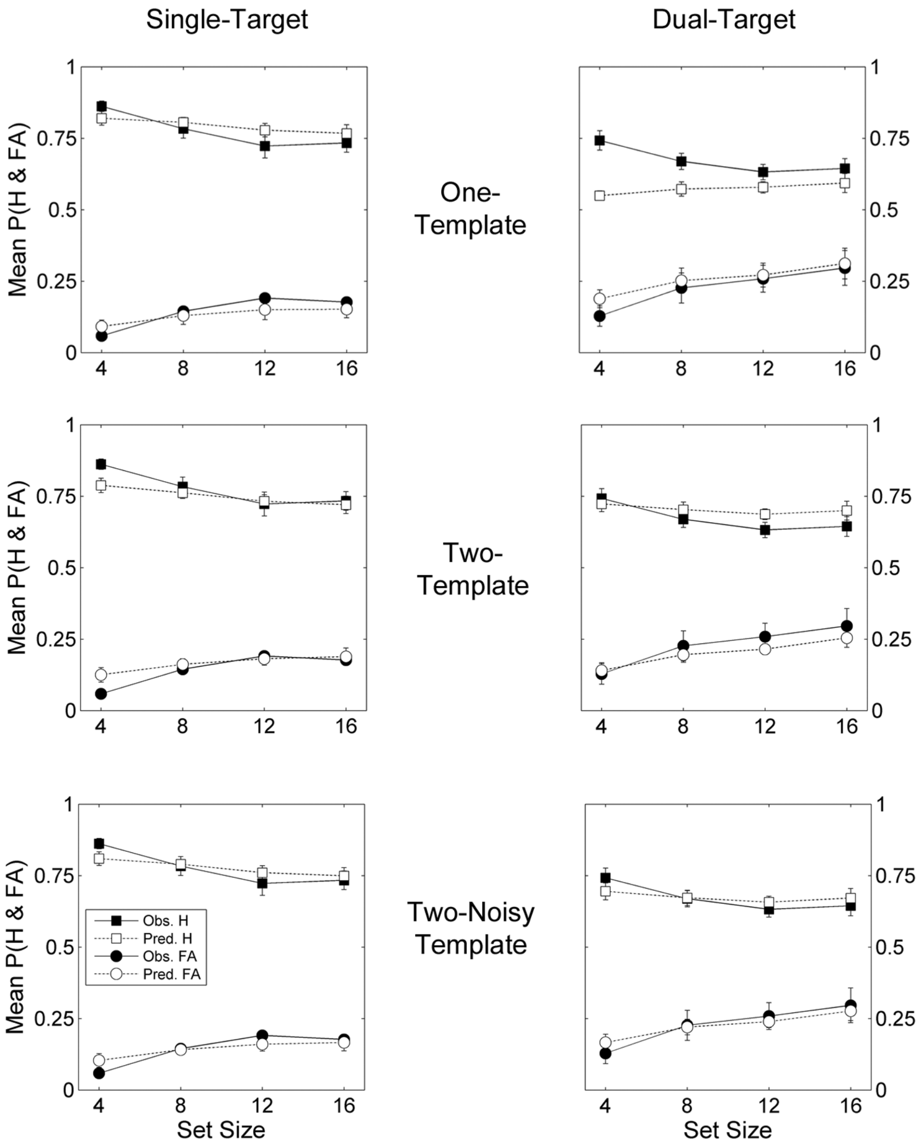

Figure 2. Observed (black symbols) and predicted (white symbols) accuracy by search type and set size for each model. Square symbols represent mean hit rates and circles represent mean false alarm rates. Error bars denote standard errors of the means.
doi:10.1371/journal.pone.0086848.g002

Table 3. Model comparison: mean estimated d′, response criterion (λ) and summed AIC values for the one-template (1-T), two-template (2-T) and two-noisy-template models (2-N-T) for single-target (sTgt) and dual-target (dTgt) searches.

			λ				
**Model**	**Search**	***d′***	**4**	**8**	**12**	**16**	**Σ AIC**
1-T	sTgt	3.11	2.17	2.32	2.44	2.46	1445.09
	dTgt		1.72	1.91	2.05	2.06	
2-T	sTgt	2.73	1.95	2.13	2.27	2.31	1260.35
	dTgt		2.23	2.39	2.50	2.50	
2-N-T	sTgt	2.95	2.07	2.23	2.35	2.40	1219.61
	dTgt	2.53	2.15	2.34	2.44	2.45	

doi:10.1371/journal.pone.0086848.t003

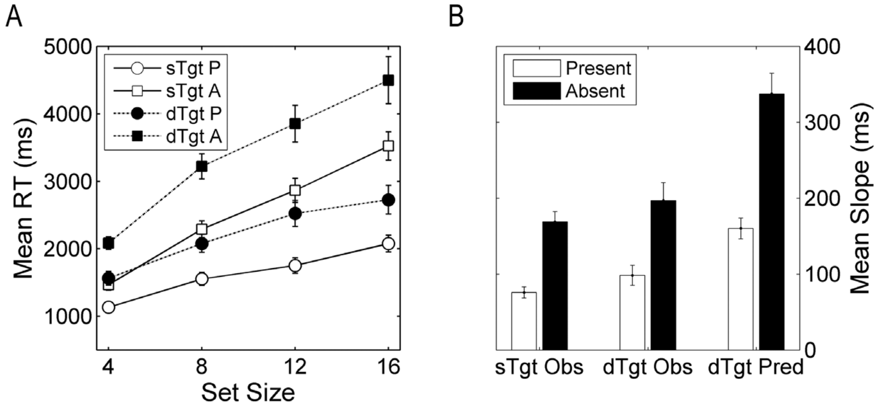

Figure 3. Mean RT by trial type and set size on single- and dual-target searches (a) and Mean observed RT-by-set-size slopes on single- (sTgtObs) and dual-target (dTgt Obs) searches and predicted RT-by-set-size slopes (dTgt Pred) for two consecutive single-target searches (b). Error bars denote standard errors of the means.
doi:10.1371/journal.pone.0086848.g003

A second 2×2 ANOVA on the RT-by-set-size intercepts with search type and trial type as within-subjects factors yielded a main effect of search type [$F(1,13) = 45.48$, $p<0.001$, $\eta_p^2 = 0.78$], no effect of trial type [$F(1,13) <1$], and a significant Search type by Trial type interaction [$F(1,13) = 6.55$, $p = 0.02$, $\eta_p^2 = 0.034$]. The main effect of search type reflected higher intercepts on dual- compared to single-target searches ($M = 483.06$ ms, $SD = 189.52$ ms) and the significant interaction was driven by differences between target-present versus target-absent trials on single- and dual-target searches respectively (see Table 4). Post hoc tests showed that the neither of these differences was statistically significant (*p*s >.1).

The results, therefore, reveal a large increase in the time required to conduct dual- compared to single-target searches that manifests primarily as an increase in the intercepts on dual-target searches. RT-by-set-size slopes also showed a modest increase on dual-target searches, but this was much smaller than that predicted by two consecutive, self-terminating single-target searches.

Discussion

The current findings replicate the dual-target costs observed in a number of previous studies [1–4], [9]. In order to elucidate the mechanism underlying these costs, we sought to distinguish between the serial and parallel accounts by investigating three questions: (a) is dual-target search characterised by a reduction in the amount of information available to guide search, (b) does any decrease in accuracy on dual-target searches exceed that predicted by two independent single-target searches and (c) is the increase in RTs as a function of set size in dual-target search consistent with two consecutive single-target searches?

To investigate the first two questions, we contrasted the observed probability distributions of hits and false alarms with those predicted by three SDT models: a one-template, two-template and two-noisy-template model. AIC values were largest for the one-template model, indicating a worse fit between the observed and predicted rates of accuracy than for either of the two-template models. For all but two observers, hit rates on dual-target searches were higher than predicted by the one-template model. False alarm rates also tended to be lower than those predicted, indicating observers were able to use information from both target-templates to detect or reject the presence of a target in the display. Except for two observers at a set size of 16, false alarm rates were also lower than chance, ruling out the possibility that observers simply guessed when the target matching the active target-template was absent in the display. AIC values were also higher for the two-template than the two-noisy-template model, supporting a reduction in *d'* on dual- compared to single-target searches. Although this was small (*M* reduction in *d'* = 0.42), these results suggest the dual-target cost in accuracy can be attributed to three factors: (a) an increase in stochastic noise on dual- compared to single-target searches, (b) changes in the observers' response criteria as the number of potential targets increases and (c) a decrease in the quality of the information used to classify objects in the display when two targets are sought.

The analyses above provide information about the number of target-templates used to guide dual-target search but are uninformative with respect to the time-course of their activation. Comparisons of RT-by-set-size functions, however, provide a means of evaluating this information against the predictions of serial and parallel models of dual-target search. According to the serial model, attentional control at any one time is limited to a single target-template, [12], [13], [16]. This predicts a slowing of dual- compared to single-target searches, because the observer will need to activate both target-templates to find or reject the target on half the trials. Our data revealed a significant increase in RT-by-set-size slopes on dual- compared to single-target searches. Much of this appears to reflect a larger rate of change on dual-compared to single-target searches between set sizes of four and

Table 4. Regression statistics for mean RT-by-set-size functions by search and trial type. Intercept and slope values are reported in milliseconds and milliseconds per item respectively.

	Slope	Intercept	*F* value	r^2	*p* value
Single-target present p_1	76	870	375.07	0.99	0.002
Single-target absent a_1	168	848	111.26	0.98	0.001
Dual-target present p_2	98	1238	59.78	0.97	0.012
Dual-target absent a_2	196	1446	83.71	0.96	0.012

doi:10.1371/journal.pone.0086848.t004

eight, particularly on target-absent trials (see Figure 3a). Moreover, the increase in RT-by-set-size slopes on dual- compared to single-target searches was much smaller than that predicted by two consecutive, single-target searches. Assuming RT-by-set-size slopes on single-target trials provide an index of the time required to compare objects in the display against the target-template in VWM, the increase in dual-target slopes equates to only 58% of the time required to conduct a second, single-target search. At a set size of four, this means observers would have time to compare less than one of the additional four objects required to detect or reject the presence of both targets. This finding replicates those observed during single- and dual-target searches for more complex stimuli [3] and suggests observers conduct parallel rather than consecutive single-target searches when two targets are sought.

The modest increase in the slopes on dual- compared to single-target searches contrasts with the large increase in the intercepts of the RT-by-set-size functions: dual-target intercepts were approximately double those on single-target searches. This general slowing of RTs has been observed in previous studies investigating the impact of VWM load on the time-course of search. Woodman and colleagues [35] reported an increase in the intercept but not the slope of RT-by-set-size functions when observers were required to remember the identity of four objects during a single-target search. They interpreted this as evidence that VWM load affects processes that occur before and after, but not during search, such as the instantiation of the target-template and response selection. More recently, however, Solomon and colleagues [25] have shown that VWM load during search can decrease the accuracy of fixations without affecting the slope of the RT-by-set-size functions. This reduction, which was characterised by an increase in the probability of fixating a distractor as well as the likelihood of regressive saccades, is easily reconciled with our own results: first, the decrease in *d'* is consistent with a reduction in the quality of the information available to guide eye movements as VWM load increases from one to two target-templates and second, the requirement to access information from multiple target-templates has a limited effect on the rate of search through the display.

The combination of RT and SDT analyses in the current study provides complementary evidence about the time-course and the amount of information used to guide dual-target search. SDT models are usually used to characterise search processes within a single fixation. Studies investigating search as a function of set size typically present objects at fixed eccentricities in brief displays ($<$100 ms) to control for the effects of eye movements and crowding on target discriminability [12], [19], [36]. In the current study, we have generalised models derived in these highly controlled experiments to the free-view conditions more typically experienced during real-world searches. Changes in decision criteria as set size increases in our data, therefore, may be attributed in part to sensory factors such as spatial acuity and lateral inhibition [18], [37–38]. Importantly, however, these do not vary across single- and dual-target searches, where the only difference between the objects in the display is their relationship to the target-templates in VWM. The increased likelihood of the two-noisy-template model for our data, therefore, supports a reduction in the quality of the information available to identify targets when observers activate two target-templates during search. Increases in RTs have previously been linked to decreases in target discriminability [39], providing an explanation for the small increase in the RT-by-set-size slopes on dual- compared to single-target searches. The majority of the dual-target cost in RTs, however, appears to reflect an increase in the time required to initiate attentional guidance and select the correct response, rather than a slowing of the search process itself [25], [35].

The results of the current study are consistent with a parallel model of dual-target search [10–11], [21]. The requirement to maintain two target-templates, however, appears to impose a reduction in the quality of the information available to guide selection and categorise objects in the display as targets or distractors. Recent findings have revealed an inverse relationship between the number of to-be-remembered objects and the precision with which they are maintained in VWM [22–24]. Generalising this to search predicts a decrease in the precision of the target-templates in dual- compared to single-target searches and a corresponding reduction in the signal to noise ratio that determines target discriminability. An alternative explanation is that competition between simultaneously active target-templates either degrades the representation of each target in VWM, or requires observers to adopt a strategy optimised to incorporate both sources of information during search. This might entail fixations designed to minimise the foveal distance between separate objects for multiple comparisons rather than single objects most likely to be one target or the other. In the former explanation, the reduction in accuracy would reflect a change in the VWM representations that guide search. In the latter, the reduction would be attributable to a change in the quality of the information obtained during eye movements. While neither explanation is mutually exclusive, the possibility that the reduction in target discriminability reflects a strategic change in saccadic sampling poses an interesting question about the way observers transform top-down biases from simultaneously active target-templates during search into a sequence of serial fixations.

The current results provide information about the mechanisms underlying the dual-target cost in free-view displays. Evidence for parallel, capacity limited search in our data, however, does not preclude a serial mode of dual-target search in other situations [12], [16–17]. In our study, targets defined by variation along a single feature-dimension were matched in terms of the accuracy and speed with which they were detected. Differences in the discriminability of the cued targets in other studies may have elicited a serial strategy of dual-target search that prioritised the most salient or memorable object first [12]. In this case, variability in the results across different tasks may reflect a flexible relationship between the information in VWM and attentional control during search, rather than support for one or other exclusive accounts of the dual-target cost. Despite this, there is increasing evidence that the requirement to compare perceptual input with multiple items in VWM can degrade the comparison process independently of whether these are active at the same or different times. In a recent study, Woodman and Vecera [40] found that withdrawing attention from one VWM representation to activate another results in the decay of the unattended memorandum (see also [1]). In their task, the accuracy of recall for features belonging to the same or different objects was tested serially. In the current study, a similar conclusion can be drawn on the basis that the requirement to distribute attention across multiple target-templates results in the partial withdrawal of resources from both. Whether the resulting reduction in target discriminability occurs during the encoding or maintenance of the cues, or the search process itself, has yet to be determined. Furthermore, the ways strategic and stimulus-driven factors interact to determine how and when attentional control during search is distributed across items in VWM remain important issues for further research.

Supporting Information

Figure S1 Individual P values for a X^2 goodness-of-fit test for the 1-Template, 2-Template and 2-Noisy-Template models of single- and dual-target search.
(TIF)

Acknowledgments

Thanks to Alan Palmer at the MRC's Institute of Hearing Research and Gordon Harold at the School of Psychology, University of Sussex, for their support during the preparation of the manuscript. Thanks also to Mark Lansdale for helpful comments on an early version of the draft and to Graham Hutton, for allowing discussions about SDT to disrupt numerous coffees at Le Mistral.

Author Contributions

Conceived and designed the experiments: DB. Performed the experiments: DB. Analyzed the data: DB OZ. Contributed reagents/materials/analysis tools: DB OZ. Wrote the paper: DB.

References

1. Menneer T, Barrett DJK, Phillips L, Donnelly N, Cave KR (2007) Costs in searching for two targets: dividing search across target types could improve airport security screening. Appl Cogn Psychol 21: 915–932.
2. Stroud MJ, Menneer T, Cave KR, Donnelly N (2012) Using the dual-target cost to explore the nature of search representations. J Exp Psychol Hum Percept Perform 8: 113–122.
3. Menneer T, Cave KR, Donnelly N (2009) Cost of Search for Multiple Targets: The Effects of Practice and Target Similarity. J Exp Psychol Appl 15: 125–139.
4. Godwin HJ, Menneer T, Cave KR, Helman S, Way RL, et al. (1010) The impact of relative prevalence on dual-target search for threat items. Acta Psychol 134: 79–84.
5. Bundesen C (1990) A theory of visual attention. Psychol Rev 97: 523–547.
6. Duncan J, Humphreys GW (1989) Visual search and stimulus similarity. Psychol Rev 96: 433–458.
7. Desimone R, Duncan J (1995) Neural mechanisms of selective visual attention. Annu Rev Neurosci 18: 193–222.
8. Carlisle NB, Arita JT, Pardo D, Woodman GF (2011) Attentional templates in visual working memory. J Neurosci 31: 9315–9322.
9. Menneer T, Stroud MJ, Cave KR., Li X, Godwin HJ, Liversedge SP, Donnelly N (2012) Search for two categories of target produces fewer fixations to target-color items. J Exp Psychol Appl 18: 404–418.
10. Beck VM, Hollingworth A, Luck SJ (2012) Simultaneous control of attention by multiple working memory representations. Psychol Sci 23: 887–898.
11. Roper ZJ, Vecera SP (2012) Searching for two things at once: Establishment of multiple control settings on a trial-by-trial basis. Psychon Bull Rev 19: 1114–1121.
12. Houtkamp R, Roelfsema PR (2009) Matching of visual input to only one item at any one time. Psychol Res 73: 317–326.
13. Olivers NL, Peters J, Houtkamp R, Roelfsema PR (2011) Different states in visual working memory when it guides attention and when it does not. Trends Cogn Sci 15: 327–334.
14. McElree B (2001) Working memory and focal attention. J Exp Psychol Learn Mem Cogn 27: 817–835.
15. Oberauer K (2002) Access to information in working memory: Exploring the focus of attention. J Exp Psychol Learn Mem Cogn 28: 411–421.
16. Gavavan H (1998) Serial attention within working memory. Mem Cognit 26: 263–276.
17. Sternberg S (1969) Memory-scanning: Mental processes revealed by reaction-time experiments. Am Sci 57: 421–457.
18. Eckstein MP (2011) Visual search: A retrospective. J Vis 11: 14, 1–36.
19. Palmer J (1994) Set-size effects in visual search: the effect of attention is independent of the stimulus for simple tasks. Vision Res 34: 1703–1721.
20. Wilken P, Ji Ma W (2004) A detection theory account of change detection. J Vis 4: 1120–1135.
21. Irons JL, Folk CL, Remmington RW (2012) All set! Evidence of simultaneous attentional control settings for multiple target colors. J Exp Psychol Hum Percept Perform 38: 758–775.
22. Bays PM, Husain M (2008). Dynamic shifts of limited working memory resources in human vision. Science 321: 851–854.
23. Burmester A, Wallis G (2012) Contrasting predictions of low- and high-threshold models for the detection of changing visual features. Perception 41: 505–516.
24. Salmela VR, Saarinen J (2013) Detection of small orientation changes and the precision of working memory. Vision Res 76: 17–24.
25. Solomon GJF, Cheyne JA, Smilek D (2011). Memory load affects visual search processes without influencing search efficiency. Vision Res 51: 1185–1191.
26. Caspi A, Beutter BR, Eckstein M (2003) The time-course of visual information accrual guiding eye movment decisions. Proc Natl Acad Sci U S A 101: 13086–13090.
27. Brainard DH (1997) The Psychophysics Toolbox. Spat Vis 10: 433–436.
28. Wheeler ME, Treisman AM. (2002) Binding in short-term visual memory. J Exp Psychol Gen 131: 48–64.
29. Shaw M (1982) Attending to multiple sources of information I. The integration of information in decision making. Cogn Psychol 14: 353–409.
30. Smith PL (2010) Spatial attention and the detection of weak visual signals. In: Coltheart V editor. Tutorials in Visual Cognition. London UK: Psychology Press Ltd. 211–260.
31. Verghese P (2001) Visual search and attention: A signal detection theory approach. Neuron 31: 523–535.
32. Akaike H (1974) A new look at the statistical model identification. IEEE Trans Automat Contr AC-19: 716–723.
33. Tollenaar N, Mooijart A (2003) Type I errors and power of the parametric goodness-of-fit test. Full and limited information. Br J Math Stat Psychol 56: 271–288.
34. Wolfe JM (1998). Visual search. In: Pashler H, editor. Attention. Hove, East Sussex, UK: Psychology Press Ltd. 13–74.
35. Woodman GF, Vogel EK, Luck SJ (2001) Visual search remains efficient when visual working memory is full. Psychol Sci 12: 219–224.
36. Cameron EL, Tai JC, Eckstein MP, Carrasco M (2004) Signal detection theory applied to three visual search tasks – identification, yes/no detection and localization. Spat Vis 17: 259–325.
37. Carrasco M, Evert DL, Chang I, Katz SM (1995) The eccentricity effect: Target eccentricity affects performance on conjunction searches. Percept Psychophys 58: 1241–1261.
38. Carrasco M, Yeshurun Y (1998) The contribution of covert attention to the set-size and eccentricity effects in visual search. J Exp Psychol Hum Percept Perform 24: 673–692.
39. Palmer J (1998) Attentional effects in visual search relating search accuracy and search time. In: Wright R, editor. Visual Attention. New York: Oxford University Press. 295–306.
40. Woodman GF, Vecera SP (2011) The cost of accessing an object's features stored in visual working memory. Vis cogn 19, 1–12.
